# Inflammation and vascular permeability correlate with growth in sporadic vestibular schwannoma

**DOI:** 10.1093/neuonc/noy177

**Published:** 2018-11-02

**Authors:** Daniel Lewis, Federico Roncaroli, Erjon Agushi, Dominic Mosses, Ricky Williams, Ka-loh Li, Xiaoping Zhu, Rainer Hinz, Ross Atkinson, Andrea Wadeson, Sharon Hulme, Helen Mayers, Emma Stapleton, Simon K L Lloyd, Simon R Freeman, Scott A Rutherford, Charlotte Hammerbeck-Ward, D Gareth Evans, Omar Pathmanaban, Alan Jackson, Andrew T King, David J Coope

**Affiliations:** 1 Manchester Centre for Clinical Neurosciences, Salford Royal NHS Foundation Trust, Manchester Academic Health Science Centre, Manchester, UK; 2 Manchester Skull Base Unit, Manchester Centre for Clinical Neurosciences, Salford Royal NHS Foundation Trust, Manchester Academic Health Science Centre, Manchester, UK; 3 Wolfson Molecular Imaging Centre, Division of Informatics, Imaging and Data Sciences, University of Manchester, Manchester, UK; 4 Division of Neuroscience and Experimental Psychology, School of Biological Sciences, Faculty of Biology, Medicine and Health, University of Manchester, Manchester, UK; 5 Brain Tumour Biobank, Salford Royal NHS Foundation Trust, Manchester Academic Health Science Centre, Manchester, UK; 6 Department of Cellular Pathology, Salford Royal NHS Foundation Trust, Manchester Academic Health Science Centre, Manchester, UK; 7 Manchester Centre for Genomic Medicine, St Mary’s Hospital, Manchester University Hospitals National Health Service Foundation Trust and Manchester Academic Health Science Centre, Manchester, UK

**Keywords:** vestibular schwannoma, inflammation, TSPO, PET imaging, DCE-MRI

## Abstract

**Background:**

Inflammation is hypothesized to be a key event in the growth of sporadic vestibular schwannoma (VS). In this study we sought to investigate the relationship between inflammation and tumor growth in vivo using the PET tracer ^11^C-(*R*)-PK11195 and dynamic contrast enhanced (DCE) MRI derived vascular biomarkers.

**Methods:**

Nineteen patients with sporadic VS (8 static, 7 growing, and 4 shrinking tumors) underwent prospective imaging with dynamic ^11^C-(*R*)-PK11195 PET and a comprehensive MR protocol, including high temporal resolution DCE-MRI in 15 patients. An intertumor comparison of ^11^C-(*R*)-PK11195 binding potential (BP_ND_) and DCE-MRI derived vascular biomarkers (*K*^trans^, *v*_p_, *v*_e_) across the 3 different tumor growth cohorts was undertaken. Tissue of 8 tumors was examined with immunohistochemistry markers for inflammation (Iba1), neoplastic cells (S-100 protein), vessels (CD31), the PK11195 target translocator protein (TSPO), fibrinogen for vascular permeability, and proliferation (Ki-67). Results were correlated with PET and DCE-MRI data.

**Results:**

Compared with static tumors, growing VS displayed significantly higher mean ^11^C-(*R*)-PK11195 BP_ND_ (−0.07 vs 0.47, *P* = 0.020), and higher mean tumor *K*^trans^ (0.06 vs 0.14, *P* = 0.004). Immunohistochemistry confirmed the imaging findings and demonstrated that TSPO is predominantly expressed in macrophages. Within growing VS, macrophages rather than tumor cells accounted for the majority of proliferating cells.

**Conclusion:**

We present the first in vivo imaging evidence of increased inflammation within growing sporadic VS. Our results demonstrate that ^11^C-(*R*)-PK11195 specific binding and DCE-MRI derived parameters can be used as imaging biomarkers of inflammation and vascular permeability in this tumor group.

**Key Points**

1. Inflammation and vascular permeability are related to growth in sporadic vestibular schwannoma
2. PET markers of inflammation and DCE MRI permeability metrics can detect growth in sporadic VS
3. Macrophages account for the majority of proliferating cells in sporadic VS

**Importance of the study**

VS has an unpredictable natural history. Some tumors show sustained growth, while others remain stable or even shrink. An understanding of the mechanism of growth may have prognostic and predictive potential and lead to the development of novel therapeutic approaches. Previous evidence based on tissue analysis suggested that inflammation and angiogenesis may be relevant to VS growth, but no in vivo studies have ever been performed. Using an established PET tracer for inflammation and DCE-MRI derived vascular biomarkers, we have provided the first in vivo evidence that increased inflammation and vascular permeability correlate with growing VS. The results of this study suggest further investigation of these imaging biomarkers as predictors of tumor growth, and consideration of inflammation as a therapeutic target in sporadic VS.

Vestibular schwannoma (VS) is a benign tumor arising from the vestibular component of the VIII cranial nerve. It accounts for approximately 8% of intracranial tumors in adults, with an incidence of 1 per 100000^1^ and occurs most commonly as a sporadic lesion. Sporadic VS is thought to be caused by inactivation of the *NF2* gene and loss of the suppressor protein merlin.^2^ Although regarded as a benign tumor in the World Health Organization (WHO) classification, VS can cause significant morbidity in terms of both the tumor itself and the treatment thereof. A specific challenge in the management of VS is that only about one-third of sporadic VS tumors grow, while two-thirds of them remain stable or less commonly shrink.^3^ At present there is no method for identifying which tumors will grow and which will not. Treatment options include therefore conservative management of static tumors and surgery or radiotherapy (often stereotactic radiosurgery [SRS]) for growing tumors. Growing tumors, left untreated, will eventually produce brainstem and cerebellar compression, cranial nerve dysfunction, and hydrocephalus, with potential risks including trigeminal and bulbar dysfunction, stroke, blindness, and death. Treatment-related morbidity, which includes this same list but in addition the specific risk of facial nerve paralysis, is less when tumors are diagnosed and treated while they remain small.^4^ SRS is generally accepted to be a treatment option only for tumors smaller than 3cm in diameter.^4^ Predicting growth in individual VS at presentation would enable early intervention and therefore optimize outcomes.

The mechanisms that trigger and maintain growth in VS remain unclear. Previous studies have shown a poor association between classical markers of neoplasia such as cellular proliferation indices and tumor growth.^5^ While cyst formation may contribute to volume increase in some instances,^6^ it has been suggested that inflammation and angiogenesis may also play a pivotal role.^5,7^ In neurofibromatosis type II (NF2)–associated VS, angiogenesis has proven to be sufficiently critical to tumor growth that it provides a specific therapeutic target with demonstrable response to the anti-angiogenic agent bevacizumab.^8^ Angiogenesis may also play a role in sporadic VS growth^9–11^ with vascular endothelial growth factor expression and other angiogenic factors correlating with microvessel density,^10^ tumor volume,^10^ and tumor growth rate.^9,12^ These studies, however, are inevitably based on tissue specimens. As such, their generalizability to nongrowing tumors that do not typically undergo surgery is less certain. There is therefore a need for an in vivo metric such as an imaging biomarker to enable characterization of all VS patient groups and to facilitate longitudinal studies.

The immune response is integral to growth and invasion in malignant tumors,^13^ but the role of inflammation in benign neoplasms including VS has been less widely investigated. Macrophages are commonly found in VS especially within Antoni B areas,^14^ and their presence correlates with duration of symptoms,^11^ tumor size,^5^ and rapid growth.^7^ Detection of inflammation with positron emission tomography (PET) has been the subject of considerable research, including the development of several PET ligands targeting the 18 kDa translocator protein (TSPO).^15^ The full range of TSPO function is unknown, but the molecule is expressed by inflammatory cells and its level increases significantly following their activation.^15–17^ TSPO expression has not been previously studied in sporadic VS.

Dynamic contrast enhanced (DCE) MRI non-invasively quantifies tissue microvascular structure by measuring the pharmacokinetics of intravenously administered gadolinium-based contrast agents (GBCAs).^18,19^ GBCA concentration can be quantified and modeled through approaches such as the extended Tofts model^20^ to derive a number of key tissue microvascular parameters, including *K*^trans^, *v*_p_, and *v*_e_. Whereas *v*_p_ and *v*_e_ represent the proportion of each image voxel occupied by blood plasma and extravascular-extracellular space (EES), respectively, the volume transfer constant *K*^trans^ is a composite parameter reflecting tissue blood flow, vessel surface area, and vessel permeability.^19,20^ While a limitation in the widespread usage of DCE-MRI in clinical practice has been variation in acquisition protocols and modeling approaches across different studies,^18^ DCE-MRI derived parameters have been shown to be reproducible^21^ and correlate in tissue validation studies with both vascularity markers^22,23^ and histological estimates of the EES.^24,25^ Vascular biomarkers derived from DCE-MRI have been utilized in other CNS tumors, including NF2-related tumors,^26^ but their potential role in sporadic VS has not been previously studied.

We hypothesized that tumor growth in sporadic VS is driven by intratumoral inflammation and that inflammation could therefore represent a biomarker of tumor growth and a therapeutic target. We investigated inflammation using the TSPO PET ligand ^11^C-(*R*)-PK11195 and investigated vascular parameters, including vascular permeability within these tumors, using dual-injection DCE-MRI. Tissue analysis in the group of participants who subsequently underwent surgery was used to validate the PET and DCE-MR imaging results.

## Methods

### Study Participants

This was a nonrandomized unblinded prospective study. Between December 2015 and May 2017 twenty patients with sporadic VS were recruited via the supraregional Manchester Skull Base Unit multidisciplinary team meeting at Salford Royal Hospital. Nineteen patients completed the PET and core MRI acquisitions. Exclusion criteria included the use of medication likely to interfere with ^11^C-(*R*)-PK11195 binding, such as benzodiazepines or steroids. Due to the spatial resolution of the PET camera, only patients with sporadic VS larger than 7.5 mm diameter (>3× full-width half-maximum [FWHM] of the PET camera) in the cerebellopontine angle were recruited into the study. All patients underwent at least 2 MRI acquisitions to establish tumor growth; this included the study scan in 2 cases, where definite growth was evident. The study MRI scan in addition to the results of previous MR imaging were reviewed by the multidisciplinary team and tumors were classified as static, growing, or shrinking. The median length of follow-up from diagnosis was 2.44 years (range, 1.14–6.07) for the static cohort, 3.02 years (range, 0.91–5.58) for shrinking, and 0.71 years (range, 0.16–2.37) for growing tumors, reflecting the prompt decision to treat in this group. The growth classification was based primarily upon clinical decision making in these patients, with tumors classified as static or shrinking being offered a period of observation and growing tumors being recommended for microsurgery or SRS. Volumetric measurements of tumor size were made for both the study MRI scan and the preceding clinical scan to confirm different behavior between the tumor cohorts (see Supplementary Material). This project received ethical approval from the National Research Ethics Service Greater Manchester North West research ethics committee (REC reference 15/NW/0429), and informed consent was obtained for all patients.

### PET Acquisition

PET scans were performed on a dedicated high resolution research tomograph PET brain scanner (HRRT, Siemens; field of view, axial: 252 mm, trans-axial 312 mm).^27^ Dynamic data were acquired for 60 minutes after injection of a target dose of 740 MBq of [^11^C]-(*R*)-PK11195. PET images were reconstructed using an OSEM (ordered-subset expectation maximization) 3D iterative method^28,29^ and corrected for attenuation, scatter, random coincidences, dead time, and detector normalization as previously described^16,30^ (see Supplementary Material for PET acquisition and reconstruction protocol). The voxel size of the reconstructed PET images was 1.22 mm × 1.22 mm × 1.22 mm and post-reconstruction 3D Gaussian smoothing filters were applied to the resulting images with 2 mm and 4 mm FWHM kernels to reduce image noise on the voxel level.

### MRI Acquisition

MRI data were acquired on a 1.5-tesla whole body scanner using a dedicated head coil (Philips Achieva). The following axial MR images were obtained: high-resolution 3D T1-weighted (T1W) gradient echo sequence with full brain coverage (echo time [TE] 3.2 ms, repetition time [TR] 8.6 ms, slice thickness 1.2 mm) and balanced steady state gradient echo imaging of skull base (TE 250 ms, TR 1500 ms, slice thickness 0.7 mm). Fifteen patients also underwent contrast enhanced imaging, while 4 patients were unable to undergo contrast imaging due to concerns about uncertain renal function. DCE-MRI data were collected using a dual-injection technique as previously described^31^ (see Supplementary Material for DCE-MRI acquisition protocol). A postcontrast high resolution 3D T1W gradient echo sequence of the whole brain was also obtained at the end of the DCE-MRI sequence.

### PET Image Analysis

A summed PET image was generated from the 2 mm smoothed PET images and coregistered with the T1W structural images. Parametric maps of ^11^C-(*R*)-PK11195 binding potential (BP_ND_), representing the ratio of specifically bound radioligand over the nondisplaceable one in tissue at equilibrium, were calculated using the simplified reference tissue model with atlas-defined cerebellar gray matter providing a “pseudoreference” tissue input function.^16,32^ To address potential concerns about use of a reference region adjacent to the pathology under study for modeling, relative standardized uptake values (SUVs) were also calculated as the average tumor tissue activity over the final 30 minutes divided by either cerebellar gray matter (SUV_T/Gm_) or a manually defined region of centrum semiovale bilaterally (SUV_T/WM_) (see Supplementary Material). To specifically assess if changes in vascular volume across different tumors influenced derived BP_ND_ values, we also generated BP_ND_ maps using the modified simplified reference tissue model (SRTM) for vascularity (SRTMV), which takes into account the tracer activity in the vasculature^33^ (see Supplementary Material).

### Analysis of DCE-MRI

The signal intensity time curves were converted to tissue contrast concentration curves using measured values of R1_N_.^34^ Using the arterial input function derived from low-dose high-temporal (LDHT) DCE, the LDHT-DCE tissue concentration time course curves were fitted to the extended Tofts model.^20^ Estimated values of *K*^trans^, *v*_e_, and *v*_p_ were calculated on a pixel-by-pixel basis. The generated maps were coregistered to the T1W structural images.

### Delineation of Tumor Region of Interest

Tumors were manually delineated on the coregistered T1W postcontrast image to create an object mask. For the 4 patients who had undergone imaging without contrast, the tumor was delineated on the noncontrast T1W structural image. Care was taken when delineating the tumor to avoid partial volume effects from nearby structures or surrounding CSF. The object mask was projected onto the BP_ND_ parametric map derived using both the SRTM and SRTMV models for sampling the mean (BP_mean_) and maximum (BP_max_) BP_ND_ value of the entire tumor. Similarly manually defined object masks were applied to the LDHT parametric maps to allow estimation of whole tumor *K*^trans^, *v*_e_, and *v*_p_ values.

### Tissue Analysis

Tissues were fixed in 10% buffered formalin and processed to paraffin embedding. In order to compare different immunostains in the same areas, serial 5-µm sections were cut from each block. The extent of macrophage infiltrates (anti-Iba1), Schwann cells (anti-S100), TSPO expression (anti-TSPO), microvessel area (anti-CD31), vascular permeability (anti-fibrinogen), and proliferation (anti–Ki-67) was assessed quantitatively on the tissue sections of 8 tumors using immunoperoxidase immunohistochemistry following established protocols^30,35^ (see Supplementary Material for detailed tissue analysis protocols).

### Statistical Analysis

Stata v11 was used for all statistical analyses. Normality and homogeneity of variance for all individual data parameters were assessed using the Shapiro–Wilk and Levene tests, respectively. One-way ANOVA with Bonferroni correction was used to compare the parametric PET and DCE-MRI derived values across the 3 tumor growth groups (static, growing, and shrinking). For nonparametric data (eg, tumor size, annual adjusted growth rate) the Kruskal–Wallis test with adjustment for multiple comparisons was used. For comparison of parametric immunohistochemistry-derived parameters between growing and static tumors a 2-tailed *t*-test was used. The intertumor correlation between imaging and immunohistochemistry-derived parameters are reported as Pearson’s product moment correlation coefficient (r) or Spearman’s rho in the case of nonlinear associations. For linear associations, the results of linear regression are reported as adjusted R^2^ estimates on included figures.

## Results

### Patient Demographics

Demographic details of the 19 patients who completed the PET and core MRI acquisitions are detailed in Table 1. The mean age was 57.7 years (range, 25.7–80.7 y); 11 patients were female. There was no significant difference in mean age across the 3 different groups (ANOVA, *P* > 0.05; see Supplementary Material). All tumors were unilateral; 12 were right-sided. Seven patients had comorbidities, including hypertension and type II diabetes, and 1 patient was taking aspirin; no patients were taking steroids or benzodiazepines. Eight patients underwent surgical resection, 2 were treated with SRS, and 9 had no treatment and are on long-term follow-up. Eight VS tumors were classified as static, 7 as growing, and 4 as shrinking. Tumor size ranged from 0.26 to 5.71 cm^3^; the volume of growing lesions was significantly larger than static or shrinking tumors (*P* < 0.05). Confirmatory volumetric measurements demonstrated that growing tumors displayed a significantly higher annual adjusted growth rate compared with the static (0.70 vs 0.02 cm^3^/year, *P* = 0.008) and shrinking (0.70 vs −0.28 cm^3^/year, *P* = 0.006) groups (see Supplementary Material).

**Table 1:** Demographics, tumor features, and clinical outcome of 19 study participants

Patient	**Age,y**	M:F	**Other Medical Conditions**	**Size at Time of PET (cm** ^**3**^)	**MDT Classification**	**Tumor Side**	**Cystic/Solid Tumor**	**Clinical Outcome**	**Histology (if known**)
1	66.6	F	HTN, T2DM, ocular melanoma (left eye), CKD stage 3	0.26	Shrinking	R	Solid	Follow-up	NA
2	32.3	M	Nil	1.88	Growing	L	Solid	Surgery	Schwannoma, WHO grade I
3	25.7	M	Nil	3.82	Growing	R	Small posteromedial cyst	Surgery	Schwannoma, WHO grade I
4	58.8	F	T2DM, HTN	0.92	Static	R	Solid	Follow-up	NA
5	60.8	F	Nil	1.03	Static	R	Solid	Follow-up	NA
6	56.3	F	Depression	0.30	Static	R	Solid	Surgery	Schwannoma, WHO grade I
7	69.2	F	OA, anxiety	0.59	Shrinking	L	Solid	Follow-up	NA
8	26.8	M	Nil	1.92	Shrinking	L	Small cysts	Follow-up	NA
9	61.1	F	Hashimoto’s, OA, hypothyroidism, Raynaud’s, pre-diabetes	0.37	Static	R	Solid	Surgery	Schwannoma, WHO grade I
10	76.1	F	Nil	1.25	Static	L	Small anterolateral cyst	Follow-up	NA
11	74.9	M	T2DM	1.09	Growing	R	Solid	SRS	NA
12	64.6	F	Nil	0.97	Growing	R	Solid	SRS	NA
13	73.9	F	Hypothyroidism	0.80	Static	R	Solid	Follow-up	NA
14	80.7	M	BPH	0.99	Shrinking	R	Solid	Follow-up	NA
15	63.0	M	Asthma	0.38	Static	L	Solid	Surgery	Schwannoma, WHO grade I
16	58.2	M	HTN	0.73	Static	R	Solid	Follow-up	NA
17	54.3	M	HTN	3.33	Growing	L	Solid	Surgery	Schwannoma, WHO grade I
18	54.8	F	T2DM	5.71	Growing	L	Small multiple cysts	Surgery	Schwannoma, WHO grade I
19	39.0	F	Nil	2.61	Growing	R	Small multiple cysts	Surgery	Schwannoma, WHO grade I

BPH = benign prostatic hypertrophy; CKD = chronic kidney disease; HTN = hypertension; NA = not applicable; OA = osteoarthritis; SRS = stereotactic radiosurgery; T2DM = type 2 diabetes.

### PET Data

**Fig. 1:**
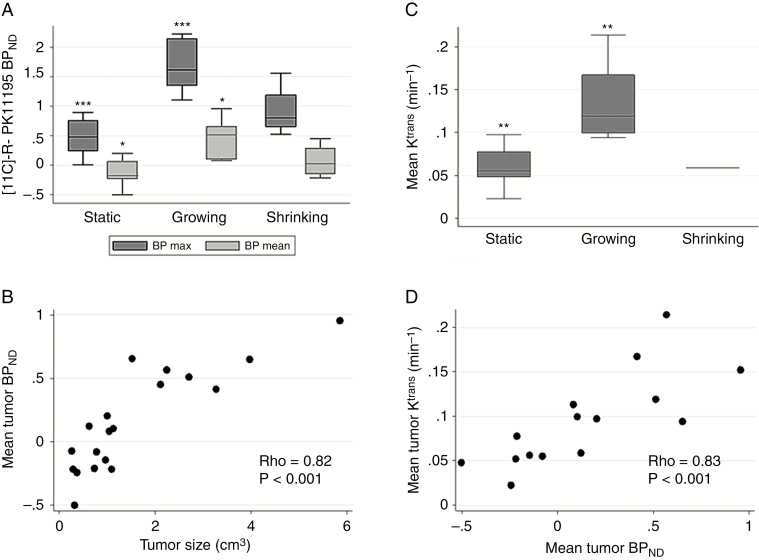
[^11^C]-(*R*)-PK11195 PET and DCE-MRI results. Boxplot of maximum and mean tumor [^11^C]-(*R*)-PK11195 BP_ND_ values categorized by MDT defined tumor growth classification (A). Intertumor scatterplot analysis of mean tumor [^11^C]-(*R*)-PK11195 BP_ND_ against tumor size (cm^3^) (B). Boxplot of mean tumor *K*^trans^ against MDT defined tumor growth classification (C). Intertumor scatterplot analysis of mean tumor *K*^trans^ against mean [^11^C]-(*R*)-PK11195 BP_ND_ for each tumor region of interest (D). One-way ANOVA with Bonferroni correction *P ≤ 0.05, **P ≤ 0.01, ***P ≤ 0.001.

Maximum and mean ^11^C-(*R*)-PK11195 BP_ND_ tumor values for the 3 growth groups are shown in Fig. 1A. Growing tumors displayed significantly higher BP_max_ (ANOVA, *P* = 0.001) and BP_mean_ (ANOVA, *P* = 0.02) compared with static tumors. SUV values similarly demonstrated that growing tumors displayed significantly higher mean SUV_T/GM_ (ANOVA, *P* < 0.01) compared with static tumors (see Supplementary Material). Correlation analysis demonstrated a positive association between derived ^11^C-(*R*)-PK11195 BP_ND_ values and both SUV_T/WM_ (r = 0.97, *P* < 0.001) and SUV_T/GM_ (r = 0.99, *P* < 0.001**).** Use of BP_ND_ values derived from the SRTMV model (see Supplementary Material) similarly demonstrated that growing tumors had significantly higher maximum (ANOVA, *P* = 0.011) and mean (ANOVA, *P* = 0.033) specific binding (BP_ND-BV_) compared with static tumors. Growing lesions also had higher BP_max_ and BP_mean_ values than shrinking tumors, but that difference was not statistically significant (ANOVA, *P* > 0.05).

Tumor size at the time of PET scanning was strongly correlated with ^11^C-(*R*)-PK11195 mean BP_ND_ (rho = 0.82, *P* < 0.001; Fig. 1B). There was no significant association between patient age or weight adjusted injected dose and ^11^C-(*R*)-PK11195 BP_ND_ within the tumor.

### DCE-MRI Data

As shown in Fig. 1C, analysis of concomitantly acquired DCE-MRI data in 15/19 patients demonstrated that growing tumors also displayed significantly higher mean tumor *K*^trans^ values than static tumors (ANOVA, *P* = 0.004). Correlation analysis of derived PET and DCE-MRI parameters demonstrated that mean tumor *K*^trans^ positively correlated with both mean BP_ND_ (rho = 0.83, *P* < 0.001; Fig. 1D) and tumor size (rho = 0.85, *P* < 0.001, see Supplementary Material). There was a moderate positive correlation of tumor size with both mean *v*_p_ (rho = 0.51, *P* = 0.05) and mean *v*_e_ (rho = 0.53, *P* = 0.04). There was no significant difference in mean tumor *v*_e_ between tumor growth groups (ANOVA, *P* > 0.1; see Supplementary Material).

### Tissue Analysis

**Fig. 2:**
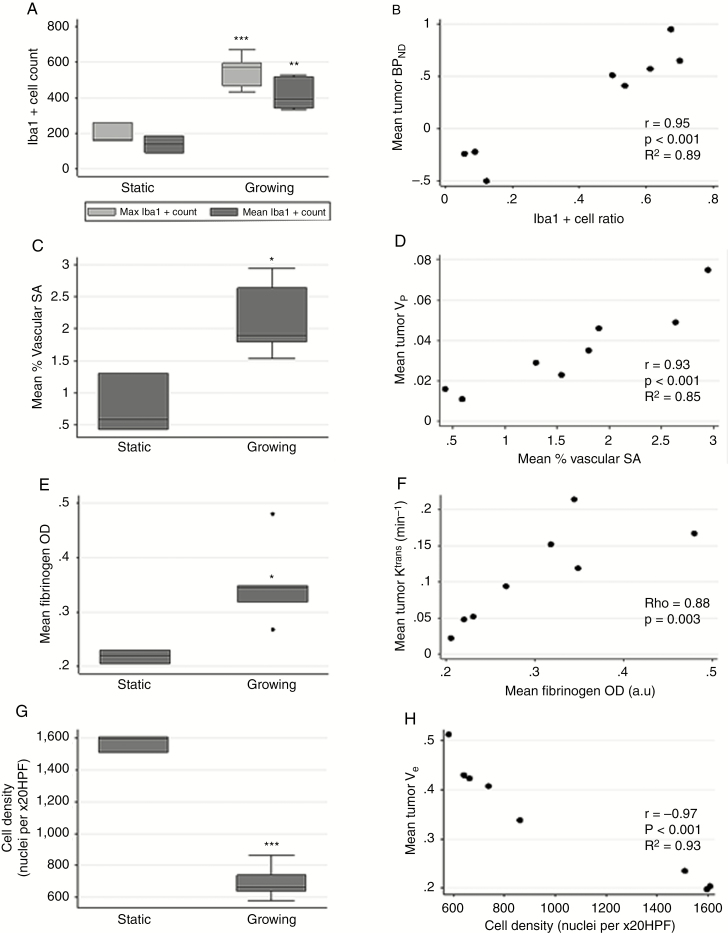
Immunohistochemistry results. Boxplot of maximum and mean tumor Iba1+ cell count per x20 high-powered field (HPF) within static and growing VS cohort (A). Intertumor scatterplot analysis of mean [^11^C]-(*R*)-PK11195 BP_ND_ against Iba1+ cell ratio (B). Boxplot of mean % vascular area (stromal area [SA]) within static and growing tumor cohort (C). Intertumor scatterplot analysis of mean tumor *v*_p_ against mean % vascular area (SA) (D). Boxplot of mean optical density (OD) measurement for fibrinogen staining within static and growing tumor cohort (E). Intertumor scatterplot analysis of mean tumor *K*^trans^ against mean fibrinogen OD (F). Boxplot of mean cell density (cell nuclei count/x20 HPF) within static and growing tumor cohort (G). Intertumor scatterplot analysis of mean cell density against mean tumor *v*_e_ (extravascular-extracellular space fraction) (H). Two-tailed *t*-test *P ≤ 0.05, **P ≤ 0.01, ***P ≤ 0.001.

**Fig. 3:**
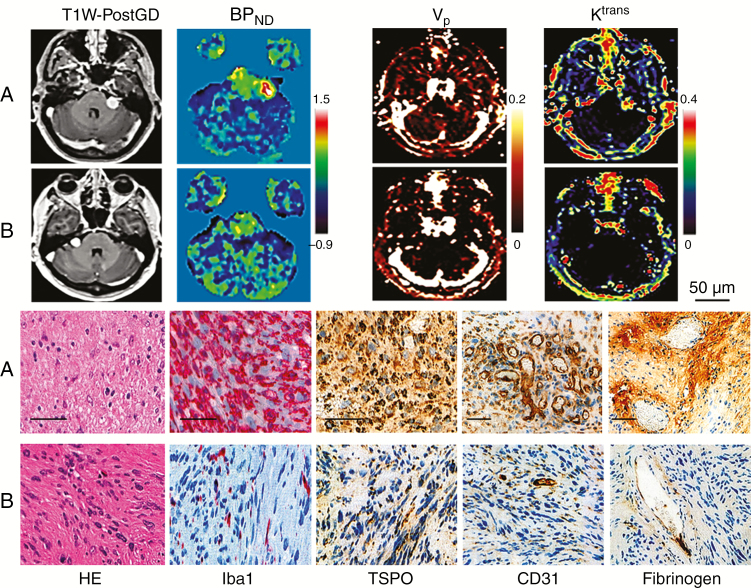
Representative images and histology from a 32-year-old male patient (*patient 2*) with a left sided growing sporadic VS (A); and a 61-year-old female (*patient 9*) with a static right-sided lesion (B).

Tumor tissue from the 8 patients who had surgery showed typical features of schwannoma with Antoni A and B areas (Fig. 3). Growing tumors displayed significantly higher maximum (2-tailed *t*-test, *P* < 0.001) and mean (2-tailed *t*-test, *P* = 0.003) Iba1+ macrophage count compared with static VS (Fig. 2A). Macrophages predominated over tumor cells in growing VS (mean Iba1+ ratio = 0.6; range, 0.5–0.7) (Fig. 3). Correlation analysis (Fig. 2B) demonstrated a strong positive association between Iba1+ cell ratio and mean tumor BP_ND_ (r = 0.95, *P* < 0.001), and between max Iba1+ cell count and max BP_ND_ (r = 0.95, *P* < 0.001). Mean TSPO optical density (OD) positively correlated with mean BP_ND_ (rho = 0.79, *P* = 0.02) (see Supplementary Material). Mean TSPO OD was higher in the growing group (*P* = 0.100), but this result was not statistically significant due to the presence of one static tumor with high TSPO OD. As shown in Fig. 2C, mean microvessel area was significantly higher in growing than static tumors (2-tailed *t*-test, *P* = 0.014) and positively correlated with DCE-MRI derived mean tumor *v*_p_ (r = 0.93, *P* < 0.001; Fig. 2D). Similarly vessel permeability seen with fibrinogen immunostaining was significantly higher in growing than static VS (2-tailed *t*-test, *P* = 0.03; Fig. 2E) and there was a strong positive relationship between derived mean tumor *K*^trans^ and fibrinogen OD (rho = 0.88, *P* = 0.003; Fig. 2F). Finally, growing tumors demonstrated significantly lower cell density (2-tailed *t*-test, *P* < 0.001; Fig. 2G) compared with static tumors, and cell density negatively correlated with mean *v*_e_ (r = −0.97, *P* < 0.001; Fig. 2H).

Upper row (from left to right): T1W post-gadolinium image, coregistered parametric map of [^11^C]-(*R*)-PK11195 BP_ND_, coregistered pixelwise maps of *K*^trans^ and *v*_p_.

Bottom row (from left to right): the hematoxylin and eosin (HE)–stained sections show the typical features of schwannoma (HE, x20); Iba1 immunostains document the macrophagic infiltrates (red, immunoperoxidase, x20), TSPO immunostains document 2 cases with high and low expression (brown, immunoperoxidase, x20), CD31 highlights vascular supply (brown, immunoperoxidase, x10); perivascular deposits of fibrinogen are seen in a case of growing VS (top panel), while no leak is present in a static VS (brown, immunoperoxidase, x10).

**Fig. 4:**
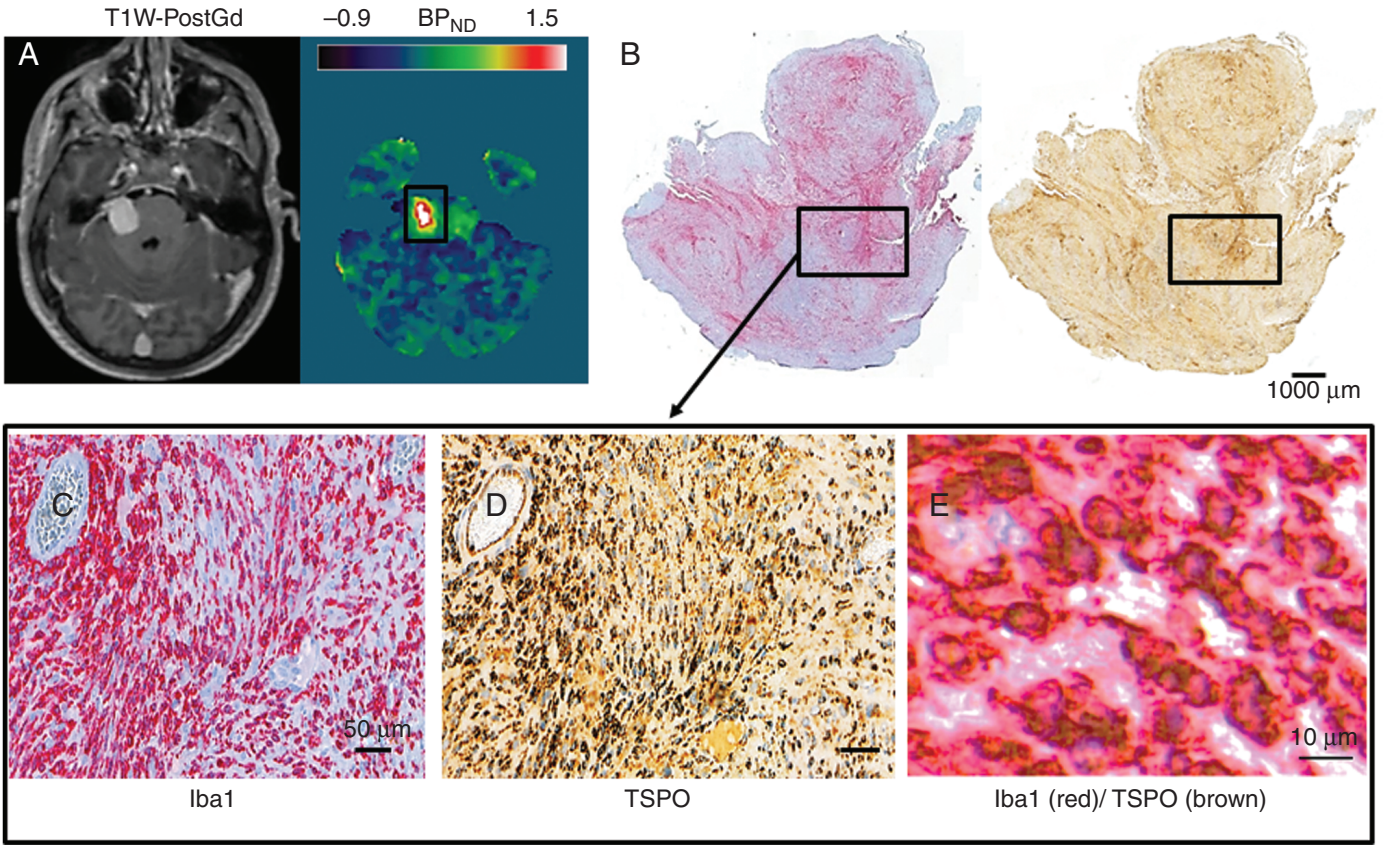
Spatial colocalization between Iba1 and TSPO in a fast growing VS.

The close relationship between immunohistochemistry and both the ^11^C-(*R*)-PK11195 PET and DCE-MRI derived parameters is shown in Fig. 3. Fig. 4 demonstrates representative imaging and immunohistochemistry from a patient with a fast growing tumor and high ^11^C-(*R*)-PK11195 specific binding. Fig. 4B-D documents the spatial correspondence between Iba1 and TSPO expression within this tumor, and double immunostaining (Fig. 4E) demonstrated colocalization of TSPO expression within the cytoplasm of Iba1+ macrophages.

Representative axial image sections (*left, T1W post-gadolinium image; right, coregistered parametric map of* [^11^C]-(*R*)-PK11195 *BP*_*ND*_) from a 25-year-old male (*patient 3*) with a right-sided rapidly growing VS (A). Immunostains (*left, Iba1; right, TSPO*) from the same tumor show the spatial correspondence between Iba1 and TSPO expression (Iba1 red, immunoperoxidase, whole mount; TSPO brown, immunoperoxidase, whole mount) (B). Higher magnification (x20 HPF) demonstrates spatial localization of Iba1 (*left*) and TSPO (*right*) from the area framed in the whole mounts (immunoperoxidase, x20) (C–D). Double immunostained (Iba1/TSPO) proves the colocalization of the 2 signals (immunoperoxidase, x40) (E).

**Fig. 5:**
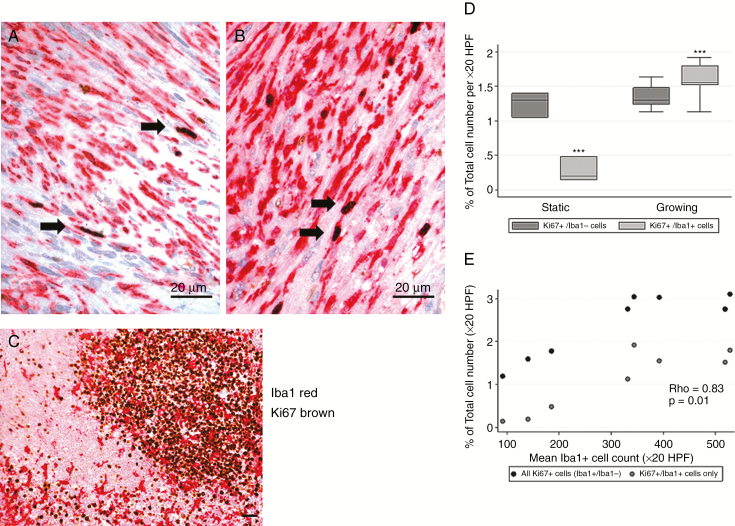
Evaluation of proliferative activity. Colocalization of Iba1 (red) and Ki-67 (brown) in a rapidly growing VS (x40 HPF) (A–B) demonstrates that inflammatory cells account for the predominant proliferative population; human tonsil is used as positive control (C) (immunoperoxidase, x20). Boxplot comparison of mean Ki-67+/ Iba1− and Ki-67*+*/Iba1+ labeling index across static and growing tumor cohort (***P < 0.001) (D). Scatterplot comparison of total Ki-67+ (*dark gray*) and Ki-67+/Iba1+ (*gray*) labeling index against mean Iba1+ cell count per x20 HPF. *Correlation coefficient (rho) for association between Ki-67+/ Iba1+ labeling index and mean Iba1+ cell count demonstrated.*

The mean Ki-67 labeling index was higher within growing tumors relative to static tumors (2.94% vs 1.52%, *P* < 0.001; see Supplementary Material). Growing tumors demonstrated a significantly higher percentage of inflammatory Ki-67+/Iba1+ cells (*P* < 0.001), which accounted for >50% of the proliferating cells within these lesions (Fig. 5). The percentage of Iba1+ cells expressing Ki-67 was similar across static and growing VS (2.91 vs 2.63%, *P* = 0.61; Supplementary Material), as was the percentage of non-inflammatory Ki-67+/Iba1− positive cells (*P* = 0.49; Fig. 5D).

## Discussion

This is the largest PET study to our knowledge in sporadic VS and the first to document a positive association between tracer uptake and tumor growth. The study demonstrated that specific binding of ^11^C-(*R*)-PK11195, a PET marker of inflammation,^36^ occurs within sporadic VS and that it is increased within growing tumors. Tissue analysis suggested that intratumoral macrophages were the major source of TSPO and therefore the PET signal, and that growing tumors were predominantly composed of inflammatory cells. Our findings suggest that inflammation may have a key role in VS growth and expand upon earlier studies which showed increased expression of macrophage surface markers within larger and fast growing tumors.^5,7^ TSPO PET imaging enables the study in vivo of inflammatory burden and characterization of those VS that would not typically be surgically resected for the first time.

^11^C-(*R*)-PK11195 has been extensively characterized as a PET tracer for neuroinflammation and less so for peripheral macrophages. Its high lipophilicity ensures that delivery is not solely dependent on endothelial permeability^16^ and use of the simplified reference tissue model in this study differentiated specific binding or binding potential (BP_ND_) from relative tracer uptake.^37^ TSPO is expressed in the endothelial and smooth muscle cells of blood vessels,^35,38^ but our tissue analysis suggested intratumoral inflammatory cells were the principal source of TSPO in growing tumors supporting the interpretation that increased ^11^C-(*R*)-PK11195 reflected increased inflammatory burden. TSPO expression was quantified using OD as a surrogate value of protein density. This approach proved more accurate in correlating PET imaging data and TSPO expression in tissue than the measurement of TSPO positive cells as it reflects the levels of TSPO available in tissue to bind the imaging probe ^11^C-(*R*)-PK11195.^30^

Recent studies have documented upregulation of pro-inflammatory cytokines such as interleukin (IL)-1β, IL-6, and tumor necrosis factor–α, and leukocyte adhesion molecules such as intercellular adhesion molecule 1 in VS compared with normal vestibular nerve.^39^ We therefore hypothesized that growing tumors express pro-inflammatory cytokines and chemokines to a critical threshold, and it is the resulting recruitment of inflammatory cells, rather than Schwann cell proliferation, which in turn leads to their continued and sometimes rapid growth. Further larger studies are needed to fully characterize these pro-inflammatory molecules and understand their respective roles in both static and growing VS.

Analysis of concomitantly acquired DCE-MRI data in 15 patients demonstrated that growing tumors displayed higher *K*^trans^ values, a measure of vascular permeability,^20,26^ and a strong association between this metric and ^11^C-(*R*)-PK11195 binding. Several methods have been proposed to evaluate vessel permeability in tissue sections, including extravascular fibrinogen assessment. Fibrinogen is a large plasma glycoprotein (340 kDa) which is only detectable in areas that have significant endothelial disruption.^40,41^ Multiple previous studies have demonstrated fibrinogen as an accurate marker of vessel permeability^40–42^ that correlates with other permeability markers such as immunoglobulin G.^41^ Supporting immunohistochemistry demonstrated both increased vascular surface area and extravascular fibrinogen staining within growing VS, suggesting a link between vascular permeability and recruitment of inflammatory cells.^43,44^

Previous studies that have investigated PET tracers such as 18-fluorodeoxyglucose or [^11^C]-methionine in sporadic VS have been inconclusive due to limited tracer uptake irrespective of tumor size or growth.^45–47^ These results may be due in part to the inherently low proliferative and thereby low metabolic activity of these tumors.^5,48^ Our data demonstrate consistent cellular proliferation rates in the neoplastic cell population irrespective of tumor behavior, with the only variation being an increased population of proliferating inflammatory cells within growing tumors. Inflammatory and vascular biomarkers may therefore be more relevant in sporadic VS than cellular proliferation.

Our study is the first to provide combined imaging and tissue demonstration of both increased inflammation and vascular permeability in growing sporadic VS. One patient in our study displayed disproportionately high TSPO expression through immunohistochemical analysis relative to the ^11^C-(*R*)-PK11195 specific binding and inflammatory cell number. The correlation between the PET assessment and inflammatory cell density was preserved and so this did not detract from the potential of ^11^C-(*R*)-PK11195 as a biomarker, but the reason for high immunohistochemical TSPO labeling in this case could not be clarified. Previous studies have shown that tumor size itself is a predictive marker of VS growth,^49^ although this is not universally accepted. Separating the relative contributions of tumor size and growth rate to ^11^C-(*R*)-PK11195 specific binding from our data is difficult as the growing tumors were also statistically significantly larger. Larger studies with dedicated follow-up imaging should be undertaken to address the prognostic benefit of ^11^C-(*R*)-PK11195 uptake or DCE derived parameters in predicting VS growth.

## Conclusion

This study presents evidence of the critical role that inflammatory cell migration plays in the growth of sporadic VS and is the first to provide an estimate of the inflammatory contribution to static tumors as well as the growing tumors that have previously been characterized ex vivo. We present a PET biomarker of inflammation alongside candidate DCE-MRI derived vascular biomarkers in the first combined study in this patient group. Our imaging and immunohistochemistry findings of both increased inflammation and vascular permeability within these tumors should prompt further research into the role these pathophysiological mechanisms play in VS growth and consideration of these processes as novel therapeutic targets.

## Funding

This project has been funded by both Cancer Research UK (CRUK) and the Engineering and Physical Sciences Research council (ESPRC) through the Cancer Imaging Centres grant (reference: C8742/A18097). FR and DM work leading to these results has received funding from the Seventh Framework Programme (FP7/2007-2013) under grant agreement nHEALTH-F2-2011-278850 (INMiND); Mr Ricky Williams has been funded by a private donation from Mr Robert Ward and his family.

## Supplementary Material

Supplementary Table 1

Supplementary Table 2

Supplementary Table 3

Supplementary Table 4

Supplementary Figure S1

Supplementary Figure S2

Supplementary Methods

Supplementary Figure Legends

## Notes

**Authorship statement.** DJC, OP, RA, ATK contributed to conception and design of this study.

DL, FR, DM, AJ, OP, ATK, and DJC contributed to the writing and review of this manuscript.

DL, OP, EA, AW, SKL, SRF, CHW, SAR, ES, DGE, and ATK contributed to patient recruitment.

DL, KL, XP, RH, EA, and DC contributed to data analysis.

FR, DL, DM, RW, HM, OP, SKL, SF, CHW, SR, and ATK contributed to tissue collection and analysis.

## References

[1] Evans DG, Moran A, King A, Saeed S, Gurusinghe N, Ramsden R Incidence of vestibular schwannoma and neurofibromatosis 2 in the North West of England over a 10-year period: higher incidence than previously thought. Otol Neurotol. 2005;26(1):93–97. 15699726 10.1097/00129492-200501000-00016

[2] de Vries M, van der Mey AG, Hogendoorn PC Tumor biology of vestibular schwannoma: a review of experimental data on the determinants of tumor genesis and growth characteristics. Otol Neurotol. 2015;36(7):1128–1136. 26049313 10.1097/MAO.0000000000000788

[3] Moffat DA, Kasbekar A, Axon PR, Lloyd SK Growth characteristics of vestibular schwannomas. Otol Neurotol. 2012;33(6):1053–1058. 22710554 10.1097/MAO.0b013e3182595454

[4] Rutherford SA, King AT Vestibular schwannoma management: what is the ‘best’ option?Br J Neurosurg. 2005;19(4):309–316. 16455536 10.1080/02688690500305399

[5] De Vries M, Hogendoorn PCW, De Bruyn IB, Malessy MJ, van der Mey AG Intratumoral hemorrhage, vessel density, and the inflammatory reaction contribute to volume increase of sporadic vestibular schwannomas. Virchows Arch. 2012;460(6):629–636. 22555941 10.1007/s00428-012-1236-9 PMC3371334

[6] Charabi S, Mantoni M, Tos M, Thomsen J Cystic vestibular schwannomas: neuroimaging and growth rate. J Laryngol Otol. 2017;108:375–379. 10.1017/s0022215100126854 8035113

[7] de Vries M, Briaire-de Bruijn I, Malessy MJ, de Bruïne SF, van der Mey AG, Hogendoorn PC Tumor-associated macrophages are related to volumetric growth of vestibular schwannomas. Otol Neurotol. 2013;34(2):347–352. 23295727 10.1097/MAO.0b013e31827c9fbf

[8] Plotkin SR, Stemmer-Rachamimov AO, Barker FG 2nd, et al Hearing improvement after bevacizumab in patients with neurofibromatosis type 2. N Engl J Med. 2009;361(4):358–367. 19587327 10.1056/NEJMoa0902579 PMC4816642

[9] Cayé-Thomasen P, Werther K, Nalla A, et al VEGF and VEGF receptor-1 concentration in vestibular schwannoma homogenates correlates to tumor growth rate. Otol Neurotol. 2005;26(1):98–101. 15699727 10.1097/00129492-200501000-00017

[10] Koutsimpelas D, Stripf T, Heinrich UR, Mann WJ, Brieger J Expression of vascular endothelial growth factor and basic fibroblast growth factor in sporadic vestibular schwannomas correlates to growth characteristics. Otol Neurotol. 2007;28(8):1094–1099. 17721409 10.1097/MAO.0b013e31814b2787

[11] Labit-Bouvier C, Crebassa B, Bouvier C, Andrac-Meyer L, Magnan J, Charpin C Clinicopathologic growth factors in vestibular schwannomas: a morphological and immunohistochemical study of 69 tumours. Acta Otolaryngol. 2000;120(8):950–954. 11200590 10.1080/00016480050218681

[12] Sass HC, Borup R, Alanin M, Nielsen FC, Cayé-Thomasen P Gene expression, signal transduction pathways and functional networks associated with growth of sporadic vestibular schwannomas. J Neurooncol. 2017;131(2):283–292. 27752882 10.1007/s11060-016-2292-9

[13] Grivennikov SI, Greten FR, Karin M Immunity, inflammation, and cancer. Cell. 2010;140(6):883–899. 20303878 10.1016/j.cell.2010.01.025 PMC2866629

[14] Wippold FJ 2nd, Lubner M, Perrin RJ, Lämmle M, Perry A Neuropathology for the neuroradiologist: Antoni A and Antoni B tissue patterns. AJNR Am J Neuroradiol. 2007;28(9):1633–1638. 17893219 10.3174/ajnr.A0682 PMC8134199

[15] Rupprecht R, Papadopoulos V, Rammes G, et al Translocator protein (18 kDa) (TSPO) as a therapeutic target for neurological and psychiatric disorders. Nat Rev Drug Discov. 2010;9(12):971–988. 21119734 10.1038/nrd3295

[16] Su Z, Herholz K, Gerhard A, et al [¹¹C]-®PK11195 tracer kinetics in the brain of glioma patients and a comparison of two referencing approaches. Eur J Nucl Med Mol Imaging. 2013;40(9):1406–1419. 23715902 10.1007/s00259-013-2447-2 PMC3738844

[17] Roncaroli F, Su Z, Herholz K, Gerhard A, Turkheimer FE TSPO expression in brain tumours: is TSPO a target for brain tumour imaging?Clin Transl Imaging. 2016;4:145–156. 27077069 10.1007/s40336-016-0168-9 PMC4820497

[18] O’Connor J, Jackson A, Parker G, Jayson G DCE-MRI biomarkers in the clinical evaluation of antiangiogenic and vascular disrupting. Br J Cancer. 2007;96:189–195. 17211479 10.1038/sj.bjc.6603515 PMC2359994

[19] Jackson A, Buckley DL, Parker GJM Dynamic contrast-enhanced magnetic resonance imaging in oncology (medical radiology/diagnostic imaging). Springer-Verlag Berlin Heidelb. 2005. doi:10.1007/b137553.

[20] Tofts PS, Brix G, Buckley DL, et al Estimating kinetic parameters from dynamic contrast-enhanced T(1)-weighted MRI of a diffusable tracer: standardized quantities and symbols. J Magn Reson Imaging. 1999;10(3):223–232. 10508281 10.1002/(sici)1522-2586(199909)10:3<223::aid-jmri2>3.0.co;2-s

[21] O’Connor JP, Jackson A, Parker GJ, Roberts C, Jayson GC Dynamic contrast-enhanced MRI in clinical trials of antivascular therapies. Nat Rev Clin Oncol. 2012;9(3):167–177. 22330689 10.1038/nrclinonc.2012.2

[22] Haris M, Husain N, Singh A, et al Dynamic contrast-enhanced derived cerebral blood volume correlates better with leak correction than with no correction for vascular endothelial growth factor, microvascular density, and grading of astrocytoma. J Comput Assist Tomogr. 2008;32(6):955–965. 19204461 10.1097/RCT.0b013e31816200d1

[23] Li X, Zhu Y, Kang H, et al Glioma grading by microvascular permeability parameters derived from dynamic contrast-enhanced MRI and intratumoral susceptibility signal on susceptibility weighted imaging head & neck imaging. Cancer Imaging. 2015;15(1):4. 10.1186/s40644-015-0039-z PMC4389664 25889239

[24] Egeland TA, Simonsen TG, Gaustad JV, Gulliksrud K, Ellingsen C, Rofstad EK Dynamic contrast-enhanced magnetic resonance imaging of tumors: preclinical validation of parametric images. Radiat Res. 2009;172(3):339–347. 19708783 10.1667/RR1787.1

[25] Aref M, Chaudhari AR, Bailey KL, Aref S, Wiener EC Comparison of tumor histology to dynamic contrast enhanced magnetic resonance imaging-based physiological estimates. Magn Reson Imaging. 2008;26(9):1279–1293. 18487033 10.1016/j.mri.2008.02.015

[26] Li KL, Djoukhadar I, Zhu X, et al Vascular biomarkers derived from dynamic contrast-enhanced MRI predict response of vestibular schwannoma to antiangiogenic therapy in type 2 neurofibromatosis. Neuro Oncol. 2016;18(2):275–282. 26311690 10.1093/neuonc/nov168 PMC4724182

[27] Sossi V, De Jong HWAM, Barker WC, et al The second generation HRRT—a multi-centre scanner performance investigation. In: IEEE Nuclear Science Symposium Conference Record. Vol 4; 2005:2195–2199.

[28] Hudson HM, Larkin RS Accelerated image reconstruction using ordered subsets of projection data. IEEE Trans Med Imaging. 1994;13(4):601–609. 18218538 10.1109/42.363108

[29] Hong IK, Chung ST, Kim HK, Kim YB, Son YD, Cho ZH Ultra fast symmetry and SIMD-based projection-backprojection (SSP) algorithm for 3-D PET image reconstruction. IEEE Trans Med Imaging. 2007;26(6):789–803. 17679330 10.1109/tmi.2007.892644

[30] Su Z, Roncaroli F, Durrenberger PF, et al The 18-kDa mitochondrial translocator protein in human gliomas: an 11C-®PK11195 PET imaging and neuropathology study. J Nucl Med. 2015;56(4):512–517. 25722450 10.2967/jnumed.114.151621

[31] Li KL, Buonaccorsi G, Thompson G, et al An improved coverage and spatial resolution using dual injection dynamic contrast-enhanced (ICE-DICE) MRI: a novel dynamic contrast-enhanced technique for cerebral tumors. Magn Reson Med. 2012;68(2):452–462. 22791559 10.1002/mrm.23252

[32] Lammertsma AA, Hume SP Simplified reference tissue model for PET receptor studies. Neuroimage. 1996;4(3 Pt 1):153–158. 9345505 10.1006/nimg.1996.0066

[33] Gunn RN, Sargent PA, Bench CJ, et al Tracer kinetic modeling of the 5-HT1A receptor ligand [carbonyl-11C]WAY-100635 for PET. Neuroimage. 1998;8(4):426–440. 9811559 10.1006/nimg.1998.0379

[34] Zhu XP, Li KL, Kamaly-Asl ID, et al Quantification of endothelial permeability, leakage space, and blood volume in brain tumors using combined T1 and T2* contrast-enhanced dynamic MR imaging. J Magn Reson Imaging. 2000;11(6):575–585. 10862055 10.1002/1522-2586(200006)11:6<575::aid-jmri2>3.0.co;2-1

[35] Veronese M, Reis Marques T, Bloomfield PS, et al Kinetic modelling of [11C]PBR28 for 18kDa translocator protein PET data: a validation study of vascular modelling in the brain using XBD173 and tissue analysis. J Cereb Blood Flow Metab. 2018;38(7):1227–1242. 28580888 10.1177/0271678X17712388 PMC6434448

[36] Venneti S, Lopresti BJ, Wiley CA Molecular imaging of microglia/macrophages in the brain. Glia. 2013;61(1):10–23. 22615180 10.1002/glia.22357 PMC3580157

[37] Gunn RN, Lammertsma AA, Hume SP, Cunningham VJ Parametric imaging of ligand-receptor binding in PET using a simplified reference region model. Neuroimage. 1997;6(4):279–287. 9417971 10.1006/nimg.1997.0303

[38] Rizzo G, Veronese M, Tonietto M, Zanotti-Fregonara P, Turkheimer FE, Bertoldo A Kinetic modeling without accounting for the vascular component impairs the quantification of [(11)C]PBR28 brain PET data. J Cereb Blood Flow Metab. 2014;34(6):1060–1069. 24667911 10.1038/jcbfm.2014.55 PMC4050251

[39] Taurone S, Bianchi E, Attanasio G, et al Immunohistochemical profile of cytokines and growth factors expressed in vestibular schwannoma and in normal vestibular nerve tissue. Mol Med Rep. 2015;12(1):737–745. 25738867 10.3892/mmr.2015.3415

[40] Gveric D, Herrera B, Petzold A, Lawrence DA, Cuzner ML Impaired fibrinolysis in multiple sclerosis: a role for tissue plasminogen activator inhibitors. Brain. 2003;126(Pt 7):1590–1598. 12805124 10.1093/brain/awg167

[41] Bridges LR, Andoh J, Lawrence AJ, et al Blood-brain barrier dysfunction and cerebral small vessel disease (arteriolosclerosis) in brains of older people. J Neuropathol Exp Neurol. 2014;73(11):1026–1033. 25289893 10.1097/NEN.0000000000000124 PMC4209852

[42] Ryu JK, McLarnon JG A leaky blood-brain barrier, fibrinogen infiltration and microglial reactivity in inflamed Alzheimer’s disease brain. J Cell Mol Med. 2009. doi:10.1111/j.1582-4934.2008.00434.x. PMC4498946 18657226

[43] Davalos D, Akassoglou K Fibrinogen as a key regulator of inflammation in disease. Semin Immunopathol. 2012;34(1):43–62. 22037947 10.1007/s00281-011-0290-8

[44] Davalos D, Ryu JK, Merlini M, et al Fibrinogen-induced perivascular microglial clustering is required for the development of axonal damage in neuroinflammation. Nat Commun. 2012;3:1227. 23187627 10.1038/ncomms2230 PMC3514498

[45] Chen JM, Houle S, Ang LC, et al A study of vestibular schwannomas using positron emission tomography and monoclonal antibody Ki-67. Am J Otol. 1998;19(6):840–845. 9831165

[46] Nyberg G, Bergstrom M, Lilja A, Muhr C, Langstrom B PET-methionine of skull base neuromas and meningiomas. Acta Otolaryngol. 1997;117:482–489. 9288200 10.3109/00016489709113425

[47] Sakamoto H, Nakai Y, Matsuda M, et al Positron emission tomographic imaging of acoustic neuromas. Acta Otolaryngol Suppl. 2000;542:18–21. 10897394 10.1080/000164800454602

[48] Gomez-Brouchet A, Delisle MB, Cognard C, et al Vestibular schwannomas: correlations between magnetic resonance imaging and histopathologic appearance. Otol Neurotol. 2001;22(1):79–86. 11314722 10.1097/00129492-200101000-00016

[49] MacKeith S, Wasson J, Baker C, et al Aspirin does not prevent growth of vestibular schwannomas: a case-control study. Laryngoscope. 2018;128(9):2139–2144. 29405309 10.1002/lary.27114

