# Supplementary material for: Inflammation and vascular permeability correlate with growth in sporadic vestibular schwannoma
Source: Neuro Oncol. 2018 Nov 2;21(3):314–25. doi: 10.1093/neuonc/noy177 (PMC6380424; doi:10.1093/neuonc/noy177)
Supplement: Supplementary Table 1 [file noy177_suppl_supplementary_table_1.docx]

**Supplementary Table 1: Comparative demographics and imaging derived parameters between static, growing and shrinking tumours**

Numbers of patients included in DCE-MRI analysis shown in brackets

|  | **Static** | **Growing** | **Shrinking** | **P value*** |
| --- | --- | --- | --- | --- |
| **N** | 8 (7) | 7(5) | 4 (1) |  |
| **Age (yrs)** | 63.5 | 49.4 | 60.8 | P>0.1 |
| **Median VS size (cm^3^)** | 0.77 | 2.61 | 0.79 | **P=0.007** |
| **Median annual adjusted growth rate**  **cm^3^/year** | 0.02 | 0.70 | -0.28 | **P=0.008** |
| **[^11^C]-(*R*)PK11195 BP_ND_ mean** | -0.07 | 0.47 | 0.07 | **P=0.02** |
| **[^11^C]-(*R*)PK11195 BP_ND_ max** | 0.57 | 1.67 | 0.92 | **P=0.001** |
| **Tumor/GM SUV ratio (SUV_T/GM_)** | 0.93 | 1.46 | 1.08 | **P=0.008** |
| **Tumour/ WM SUV ratio (SUV_T/WM_)** | 1.03 | 1.5 | 1.1 | P>0.1 |
| **Mean tumor v_p_ (no units)** | 0.03 | 0.05 | 0.03 | **P=0.08** |
| **Mean tumor K^trans^ (min^-1^)** | 0.06 | 0.14 | 0.06 | **P=0.004** |
| **Mean tumor v_e_ (no units)** | 0.36 | 0.41 | 0.37 | **P>0.1** |

** One-way ANOVA with Bonferroni correction. P value shown is for comparison between static and growing tumor cohort*
