# Supplementary material for: Inflammation and vascular permeability correlate with growth in sporadic vestibular schwannoma
Source: Neuro Oncol. 2018 Nov 2;21(3):314–25. doi: 10.1093/neuonc/noy177 (PMC6380424; doi:10.1093/neuonc/noy177)
Supplement: Supplementary Table 2 [file noy177_suppl_supplementary_table_2.docx]

**Supplementary Table 2: Correlation analysis between tumour size, [^11^C]-(*R*)-PK11195 specific binding (BP_ND_) and DCE MRI-derived parameters**

*Pearson’s product moment correlation coefficient (r) reported or Spearman’s Rho in the case of non-linear association between variables*

**Correlation analysis value using data from 15 patients who underwent combined PET and DCE-MRI

| ***Parameter*** | ***Tumour size*** | ***Mean BP_ND_***** | ***K^trans^ (min^-1^)*** | ***v_p_*** | ***v_e_*** |
| --- | --- | --- | --- | --- | --- |
| ***Tumour size (cm^3^)*** |  | **Rho= 0.91**  **P<0.001** | **Rho= 0.85**  **P<0.001** | **Rho=0.51**  **P=0.05** | **Rho=0.53**  **P=0.04** |
| ***Mean tumour* [^11^C]-(*R*)PK11195 *BP_ND_ (no units)*** | **Rho= 0.91**  **P<0.001** |  | **Rho=0.83**  **P<0.001** | Rho=0.48  P=0.07 | Rho=0.41  P=0.13 |
| ***Mean tumour***  ***K^trans^ (min^-1^)*** | **Rho= 0.85**  **P<0.001** | **Rho=0.83**  **P<0.001** |  | **Rho=0.76**  **P=0.001** | Rho=0.39  P=0.16 |
| ***Mean tumour***  ***v_p_ (no units)*** | **Rho=0.51**  **P=0.05** | Rho=0.48  P=0.07 | **Rho=0.76**  **P=0.001** |  | Rho=0.11  P=0.69 |
| ***Mean tumour***  ***v_e_ (no units)*** | **Rho=0.53**  **P=0.04** | Rho=0.41  P=0.13 | Rho=0.39  P=0.16 | Rho=0.11  P=0.69 |  |
