# Supplementary material for: Inflammation and vascular permeability correlate with growth in sporadic vestibular schwannoma
Source: Neuro Oncol. 2018 Nov 2;21(3):314–25. doi: 10.1093/neuonc/noy177 (PMC6380424; doi:10.1093/neuonc/noy177)
Supplement: Supplementary Table 3 [file noy177_suppl_supplementary_table_3.docx]

**Supplementary Table 3: Comparative immunohistochemistry derived parameters between static and growing VS**

**P value calculated using two tailed t-test*

|  | **Static** | **Growing** | **P value*** |
| --- | --- | --- | --- |
| **N** | 3 | 5 |  |
| **Mean Iba1^+^ cell count / x20HPF** | 139 | 423 | **P=0.003** |
| **Max Iba1^+^ cell count /x20HPF** | 153 | 549 | **P<0.001** |
| **Mean Iba1^+^ cell ratio** | 0.09 | 0.60 | **P<0.001** |
| **Mean TSPO OD** | 0.44 | 0.66 | **P=0.10** |
| **Max TSPO OD** | 0.55 | 0.84 | P>0.1 |
| **Mean vascular area (%)** | 0.77 | 2.16 | **P=0.014** |
| **Mean fibrinogen OD** | 0.22 | 0.35 | **P=0.03** |
| **Mean cell density (H+E cell nuclei / x20HPF)** | 1509 | 641 | **P<0.001** |
| **Mean Ki67^+^ (Iba1^+^, Iba1^-^) labelling index (%)** | 1.52 | 2.94 | **P<0.001** |
| **Mean Ki67^+^/ Iba1^-^ labelling index (%)** | 1.25 | 1.35 | P=0.49 |
| **Mean Ki67^+^/ Iba1^+^ labelling index (%)** | 0.27 | 1.58 | **P<0.001** |
| **% of Iba1^+^ cells expressing Ki67** | 2.91 | 2.63 | P=0.61 |
