# Supplementary material for: Inflammation and vascular permeability correlate with growth in sporadic vestibular schwannoma
Source: Neuro Oncol. 2018 Nov 2;21(3):314–25. doi: 10.1093/neuonc/noy177 (PMC6380424; doi:10.1093/neuonc/noy177)
Supplement: Supplementary Table 4 [file noy177_suppl_supplementary_table_4.docx]

**Supplementary Table 4: Correlation analysis of immunohistochemistry derived parameters with tumour volume, [^11^C]-(*R*)-PK11195 specific binding and dynamic contrast-enhanced MRI-derived parameters**

*** Pearson’s product moment correlation coefficient (r) reported or Spearman’s Rho in the case of non-linear association between variables*

| **Parameter** | **Mean Iba1^+^ cell count / x20HPF** | **Mean Iba1^+^ cell ratio** | **Mean TSPO OD** | **Mean percentage vascular surface area (%)** | **Mean fibrinogen OD** | **Mean cell density (HE cell nuclei / x20HPF)** | **Mean Ki67^+^/ Iba1^+^ cell labelling index (%)** |
| --- | --- | --- | --- | --- | --- | --- | --- |
| **VS size** | **Rho=0.71**  **P=0.05** | **Rho=0.88**  **P=0.004** | Rho=0.45  P=0.26 | Rho=0.53  P=0.18 | Rho=0.57  P=0.14 | **Rho= -0.90**  **P=0.002** | **Rho=0.71**  **P=0.05** |
| **Mean tumour [^11^C]-(*R*)PK11195 BP_ND_** | **r=0.84**  **P=0.009** | **r=0.95**  **P<0.001** | **Rho=0.79**  **P=0.02** | Rho-=0.53  P=0.18 | Rho=0.52  P=0.18 | **Rho= -0.71**  **P=0.05** | **r=0.84**  **P=0.009** |
| **Mean Tumour K^trans^ (min^-1^)** | **r=0.81**  **P=0.02** | r=0.80  P=0.02 | Rho=0.69  P=0.06 | **Rho=0.81**  **P=0.01** | **Rho=0.88**  **P=0.003** | **Rho= -0.71**  **P=0.05** | **r=0.93**  **P<0.001** |
| **Mean Tumour v_p_ (no units)** | **Rho=0.74**  **P=0.04** | Rho=0.53  P =0.18 | Rho=0.40  P=0.33 | **r=0.93**  **P<0.001** | **Rho=0.79**  **P=0.02** | Rho= -0.47  P=0.23 | **Rho=0.71**  **P=0.05** |
| **Mean Tumour**  **v_e_ (no units)** | Rho=0.59  P=0.12 | **r=0.91**  **P=0.002** | Rho=0.48  P=0.23 | Rho=0.60  P=0.12 | **Rho=0.74**  **P=0.04** | **r= -0.97**  **P<0.001** | **Rho=0.76**  **P=0.03** |
