# Supplementary figures and images for: Inflammation and vascular permeability correlate with growth in sporadic vestibular schwannoma
Source: Neuro Oncol. 2018 Nov 2;21(3):314–25. doi: 10.1093/neuonc/noy177 (PMC6380424; doi:10.1093/neuonc/noy177)

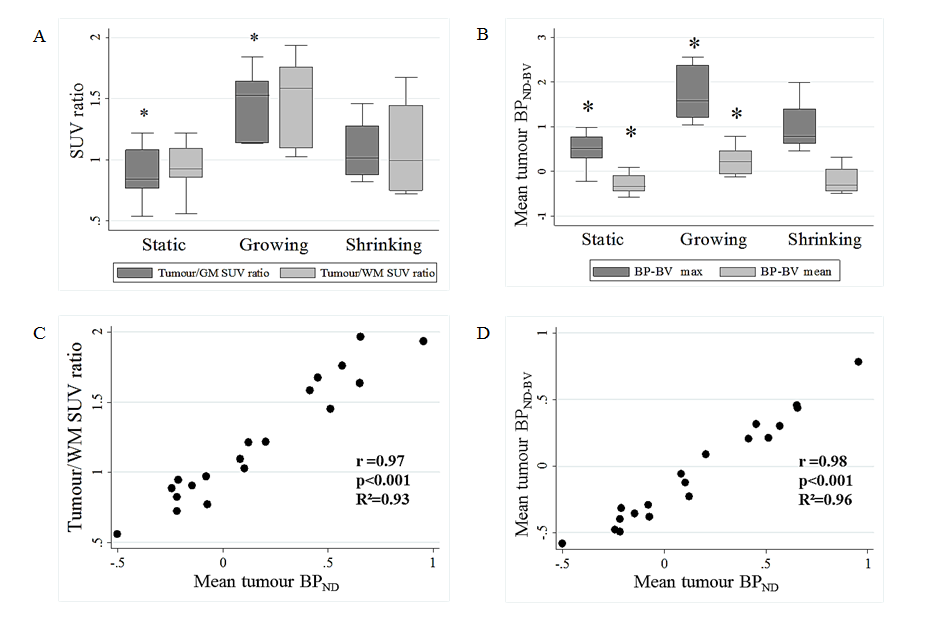

Supplement: Supplementary Figure S1 [file noy177_suppl_supplementary_figure_s1.png]

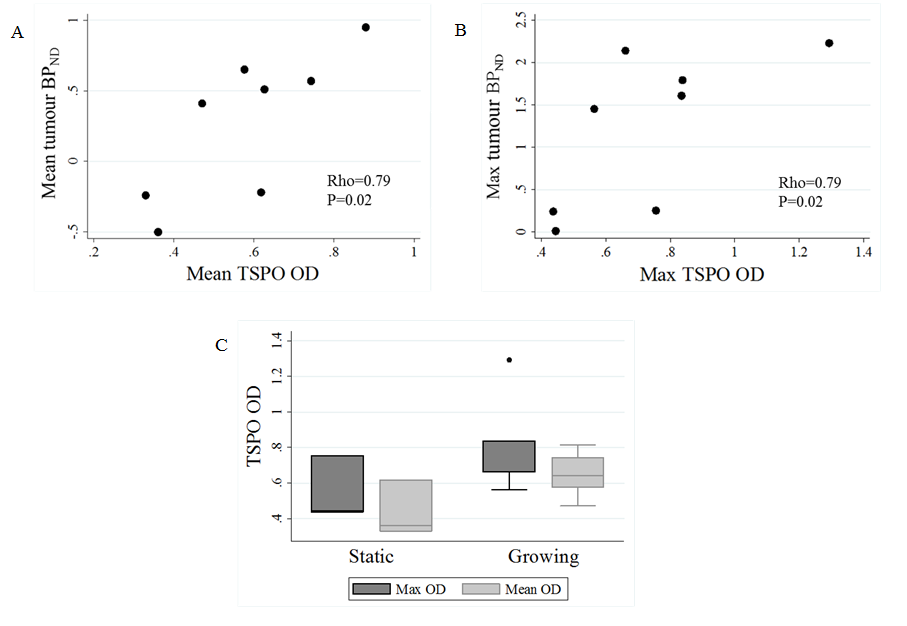

Supplement: Supplementary Figure S2 [file noy177_suppl_supplementary_figure_s2.png]
