# Supplementary material for: Inflammation and vascular permeability correlate with growth in sporadic vestibular schwannoma
Source: Neuro Oncol. 2018 Nov 2;21(3):314–25. doi: 10.1093/neuonc/noy177 (PMC6380424; doi:10.1093/neuonc/noy177)
Supplement: Supplementary Methods [file noy177_suppl_supplementary_methods.docx]

**Supplementary methods:**

**PET acquisition**

All PET scans were performed on a dedicated High Resolution Research Tomograph PET brain scanner (HRRT, Siemens; FOV, axial: 252mm, transaxial 312mm) ^27^. A 7-minute transmission scan using a Caesium^137^ point source was acquired for subsequent attenuation and scatter correction. Shortly after the start of the emission scan, a target dose of 740MBq of [^11^C]-(R) PK11195 was injected intravenously by hand as a slow bolus (10ml) over approximately 15 seconds, flushed with 0.9% saline (10ml) at the same rate. Emission data were then acquired for 60 minutes post injection. 18 time frames of increasing length were generated: one background frame of variable length prior to the injection, then one 15s frame, one 5s frame, one 10s frame, one 30s frame, four 60s frames, seven 300s frames and two 600s frames.

The multi-frame PET images were reconstructed using an ordered-subset expectation maximisation (OSEM) 3D iterative method ^28,29^. Scatter correction and corrections for random coincidences, detector normalisation, attenuation correction, and dead time required for quantification were incorporated in the reconstruction algorithm. 12 iterations with 16 subsets were used for the OSEM image reconstruction. The voxel size of the reconstructed PET images was 1.22mm×1.22mm×1.22mm and post-reconstruction 3D Gaussian smoothing filters were applied to the resulting images with 2mm and 4mm full width at half maximum (FWHM) kernels to reduce image noise on the voxel level.

**DCE-MRI acquisition protocol**

Contrast agent (CA; gadoterate meglumine; Dotarem, Geurbet S.A.) was administered by power injector as an intravenous bolus at a rate of 2 mL/s, followed by a chaser of 20 mL/s of 0.9% saline administered at the same rate. A high temporal resolution (1.0 s) sequence with a low dose of contrast agent (0.02 mmol/kg) was performed (LDHT-DCE) to allow accurate measurement of the arterial input (AIF). Subsequently, a full CA dose (0.1 mmol/kg), high-spatial resolution (voxel size = 1 × 1 × 2 mm) acquisition (FDHS-DCE) was performed to provide high spatial resolution data. Variable flip angle (VFA; α = 2°, 8°, 15° and 20°) acquisitions were performed prior to the LDHT DCE series for native longitudinal relaxation rate (R1_N_) mapping.

**Tissue analysis:**

Immunostains were performed with antibodies directed against Iba1 (microglia) (rabbit polyclonal, WAKO, Neuss Germany; dilution 1:500), anti-TSPO (goat polyclonal; Abnova, Walnut, CA USA; dilution 1:500), CD31 (endothelium) (monoclonal JC7QA, Agilent DAKO, Santa Clara, CA USA; dilution 1:30), anti-fibrinogen (monoclonal A0080, Agilent DAKO; dilution of 1:50 000), anti-Ki-67 (monoclonal M7240, Agilent DAKO, Santa Clara, CA USA; dilution of 1:50) and anti-S-100 protein (monoclonal Z0311, Agilent DAKO, Santa Clara, CA USA; dilution of 1:1000). We also performed double immunostains to co-localise Iba1 and Ki-67 and Iba1 and TSPO. Single and double immunoperoxidase immunohistochemistry was performed using the Roche Ventana BenchMark Ultra autostainer (Roche, USA) following established routine diagnostic protocols. For quantification, ten to fifteen representative images were taken at x20 magnification from HE-stained sections and sections used for single and double immunohistochemistry. TSPO, CD31 and fibrinogen images were taken using a 3D-Histech Pannoramic-250 microscope slide-scanner (3D Histech Ltd, Budapest, Hungary), HE and Ki67 stained sections were taken with a Nikon Eclipse microscope (Nikon, Kingston upon Thames, UK). Macrophages were manually counted in each tissue section by two independent observers (DL and DM) using the Kurt de vos cell counter within the Image J program (https://imagej.nih.gov/ij/) and results were then verified by a consultant neuropathologist (FR). The density of Iba1 positive cells was reported as the mean and maximum absolute cell number across ten images in addition to the mean Iba1 positive cell ratio. Cell density was quantified from HE-stained sections using a previously validated semi-automatic thresholding approach within ImageJ and was reported as the mean cell density across ten images. TSPO expression was quantified using OD as a surrogate value of protein density. This approach has proven to be more accurate in correlating PET imaging data and TSPO expression in tissue than the measurement of TSPO positive cells as it reflects the levels of TSPO available in tissue to bind the imaging probe ^34^. OD was reported as the mean and maximum values obtained across ten representative images. Microvessel area was measured in each tissue section through manual outlining of CD31 positive endothelium and reported as the mean percentage vascular surface area (SA) coverage across the ten images. Vascular permeability was determined by measuring the leak of fibrinogen^35,36^ and it was reported as the mean OD across ten representative images. Proliferation was calculated as the average Ki67 labelling percentage across 15 fields at x20 magnification (five images in each of the three highest Ki67 expressing regions).

Results of quantification were independently validated by a second observer; inter-observer agreement was greater than 95% across all measurements. All image analysis was performed on the Fiji image processing application. For OD measurements, images were deconvoluted, the red and green channels discarded, and blue channels only analysed. Thresholding of 2% for images detailing fibrinogen extra-vasation and 5% for TSPO was applied and the analyse particles tool used to quantify levels of protein expression. For analysis of CD31 expression, vessels were enlarged digitally and the outermost surface of CD31 positive vessels outlined using the freehand draw tool. Outlined areas were added to the ROI manager tool through which total area of CD31 expression in each image could be quantified. The phenotype of proliferating cells was characterised by combining Iba1 and Ki-67 immunostaining and quantified as describe above, being represented as the average ratio of the cells that were Ki67+/Iba1+. A similar approach was used to measure the number of Iba1+ cells that expressed TSPO.
