# Supplementary material for: Inflammation and vascular permeability correlate with growth in sporadic vestibular schwannoma
Source: Neuro Oncol. 2018 Nov 2;21(3):314–25. doi: 10.1093/neuonc/noy177 (PMC6380424; doi:10.1093/neuonc/noy177)
Supplement: Supplementary Figure Legends [file noy177_suppl_supplementary_figure_legends.docx]

**Supplementary Figure legends:**

**Supplementary Figure 1: SUV and SRTMV data**

Boxplot of calculated Tumour/ GM and Tumour/ WM SUV ratio categorised by MDT defined tumour growth classification (A); Boxplot of maximum and mean tumour BP_ND_  values derived using the SRTMV model (BP-BV) categorised by MDT defined tumour growth classification (B); Inter-tumor scatterplot analysis of mean tumour [^11^C]-(*R*)-PK11195 BP_ND_ against Tumour/ WM SUV ratio (C); Inter-tumor scatterplot analysis of mean tumour [^11^C]-(*R*)PK11195 BP_ND_ against mean tumour BP-BV (D)

*One-way ANOVA with Bonferroni correction * P≤0.05; ** P ≤ 0.01; ***P ≤ 0.001*

**Supplementary Figure 2: TSPO immunostaining**

Inter-tumour scatterplot analysis of mean tumour TSPO (OD ) against mean tumour [^11^C]-(*R*)-PK11195 BP_ND_ (A); Inter-tumour scatterplot analysis of maximum tumour TSPO (OD ) against maximum tumour [^11^C]-(*R*)-PK11195 BP_ND_ (B); Boxplot of mean optical density (OD) measurement for fibrinogen staining within static and growing tumour cohort (C)
